# Pregnancy Following Bariatric Surgery—Medical Complications and Management

**DOI:** 10.1007/s11695-016-2294-x

**Published:** 2016-08-03

**Authors:** Ram Prakash Narayanan, Akheel A. Syed

**Affiliations:** 1 Department of Obesity Medicine and Endocrinology, Salford Royal NHS Foundation Trust and University Teaching Hospital, Salford, UK; 2 Faculty of Medical and Human Sciences, The University of Manchester, Manchester, UK; 3 Diabetes Centre, St Helens Hospital, Marshalls Cross Road, St Helens, WA9 3DA UK

**Keywords:** Obesity, Bariatric surgery, Pregnancy, Dumping syndrome, Postabsorptive hypoglycemia

## Abstract

Bariatric surgery is most commonly carried out in women of childbearing age. Whilst fertility rates are improved, pregnancy following bariatric surgery poses several challenges. Whilst rates of many adverse maternal and foetal outcomes in obese women are reduced after bariatric surgery, pregnancy is best avoided for 12–24 months to reduce the potential risk of intrauterine growth retardation. Dumping syndromes are common after bariatric surgery and can present diagnostic and therapeutic challenges in pregnancy. Early dumping occurs due to osmotic fluid shifts resulting from rapid gastrointestinal food transit, whilst late dumping is characterized by a hyperinsulinemic response to rapid absorption of simple carbohydrates. Dietary measures are the mainstay of management of dumping syndromes but pharmacotherapy may sometimes become necessary. Acarbose is the least hazardous pharmacological option for the management of postprandial hypoglycemia in pregnancy. Nutrient deficiencies may vary depending on the type of surgery; it is important to optimize the nutritional status of women prior to and during pregnancy. Dietary management should include adequate protein and calorie intake and supplementation of vitamins and micronutrients. A high clinical index of suspicion is required for early diagnosis of surgical complications of prior weight loss procedures during pregnancy, including small bowel obstruction, internal hernias, gastric band erosion or migration and cholelithiasis.

## Introduction

Obesity affects a quarter of all adult women in Western Europe and Canada, and a third in the USA [1]. Whilst obesity in general increases the risk of various weight-related disorders, maternal obesity, defined as a BMI ≥ 30 kg/m^2^ during pregnancy, in particular increases the risk of various pregnancy complications such as miscarriage, foetal abnormality, prematurity, macrosomia, dystocia, birth injury, still birth and neonatal death, pregnancy-induced hypertension, gestational diabetes, thrombosis, difficulty in delivery leading to higher caesarean rates, anaesthetic complications, infection, postpartum haemorrhage and maternal mortality [2–5]. Whereas lifestyle and dietary measures and anti-obesity pharmacotherapy are recommended as the primary treatment approach for obesity, bariatric surgery remains the most clinically effective and cost-effective intervention for people with morbid obesity [6, 7]. Thus, the global uptake of bariatric surgery has increased exponentially in the past decade [8, 9], including in women of childbearing age [10]. Thus, pregnancy following bariatric surgery is increasingly encountered in clinical practice. These clinical presentations pose surgical, medical and obstetric challenges [2, 11, 12] requiring multidisciplinary team management. We discuss management of common complications of bariatric surgery that may be seen in pregnancy.

## Methods

We undertook a focused, non-systematic, narrative review of the literature with searches of the published literature in PubMed (www.pubmed.gov) and Google Scholar (www.scholar.google.com) with a broad range of combinations of the medical subject headings (MeSH) terms, ‘obesity’, ‘maternal obesity’, ‘bariatric surgery’, ‘gastric bypass’, ‘gastric band’, ‘sleeve gastrectomy’, ‘pregnancy’ and ‘fertility’; English language articles retrieved up to May 2015 were included.

## Results

The majority of women seeking bariatric surgery belong to the reproductive age group [13]. Thus, fertility and pregnancy are significant concerns following bariatric surgery.

### Bariatric Surgery and Fertility

Obesity in women of childbearing age is linked with lower fertility rates through associated oligo-ovulation and anovulation, even in women with regular menstrual periods [14–16]. Polycystic ovarian syndrome (PCOS) and its associated complications are also inherently linked with weight gain. Bariatric surgery can lead to prompt resolution of anovulation, improved menstrual regularity and an amelioration of PCOS linked health complications [17–20]. Overall fertility appears to improve after bariatric surgery though data is limited [21, 22]. The extent of weight loss following surgery may determine fertility potential [23]. The first 12 months after bariatric surgery represent an active catabolic state due to rapid weight loss, with gradual stabilization of the body’s nutritional state in the following months. For this reason women are generally advised to avoid pregnancy for 12–24 months after bariatric surgery [24, 25]. This aims to reduce the potential risk of intrauterine growth retardation during this period whilst allowing the woman to attain the full therapeutic benefit of the procedure [10, 26]. Pre-conception counselling in this period should include consideration of non-oral contraceptives as the efficacy of oral contraceptive pills may be compromised by unreliable absorption following bariatric surgery [27]. However, in the event that pregnancy does occur within the 12-month period following bariatric surgery, there is limited evidence that pregnancy outcomes in this situation are comparable to those of pregnancies after the 12-month period [28].

### Maternal and Foetal Outcomes

Rates of many adverse maternal and neonatal outcomes are significantly lower in women who become pregnant following bariatric surgery [4, 29, 30]. A recent large study from Sweden compared 596 singleton pregnancies in women who had previously undergone bariatric surgery with 2356 control pregnancies in women matched for pre-surgery BMI, age, parity, smoking history, educational level and delivery year [31]; this study reported lower incidences of gestational diabetes and large for gestational age babies in the postbariatric surgery women. However, rates of small for gestational age births and shorter gestation periods were higher in the postbariatric surgery women than in controls. There was no difference in rates of congenital malformation between the two groups. A recent meta-analysis of 11 cohort studies compared maternal and foetal outcomes in obese women who had undergone bariatric surgery with obese women who had not had surgery [32]. This study also reported a lower likelihood of gestational diabetes, hypertension and macrosomia following bariatric surgery but increased odds of offspring being small for gestational age; rates of caesarean section, postpartum haemorrhage and preterm delivery were not significantly different between the two groups.

### Dumping Syndromes

Dumping syndromes are common after bariatric surgery and can present diagnostic and therapeutic challenges. The pathophysiological basis of these syndromes after bariatric surgery is not well understood and maybe multifactorial [33].

#### Early Dumping Syndrome

**Fig. 1:**
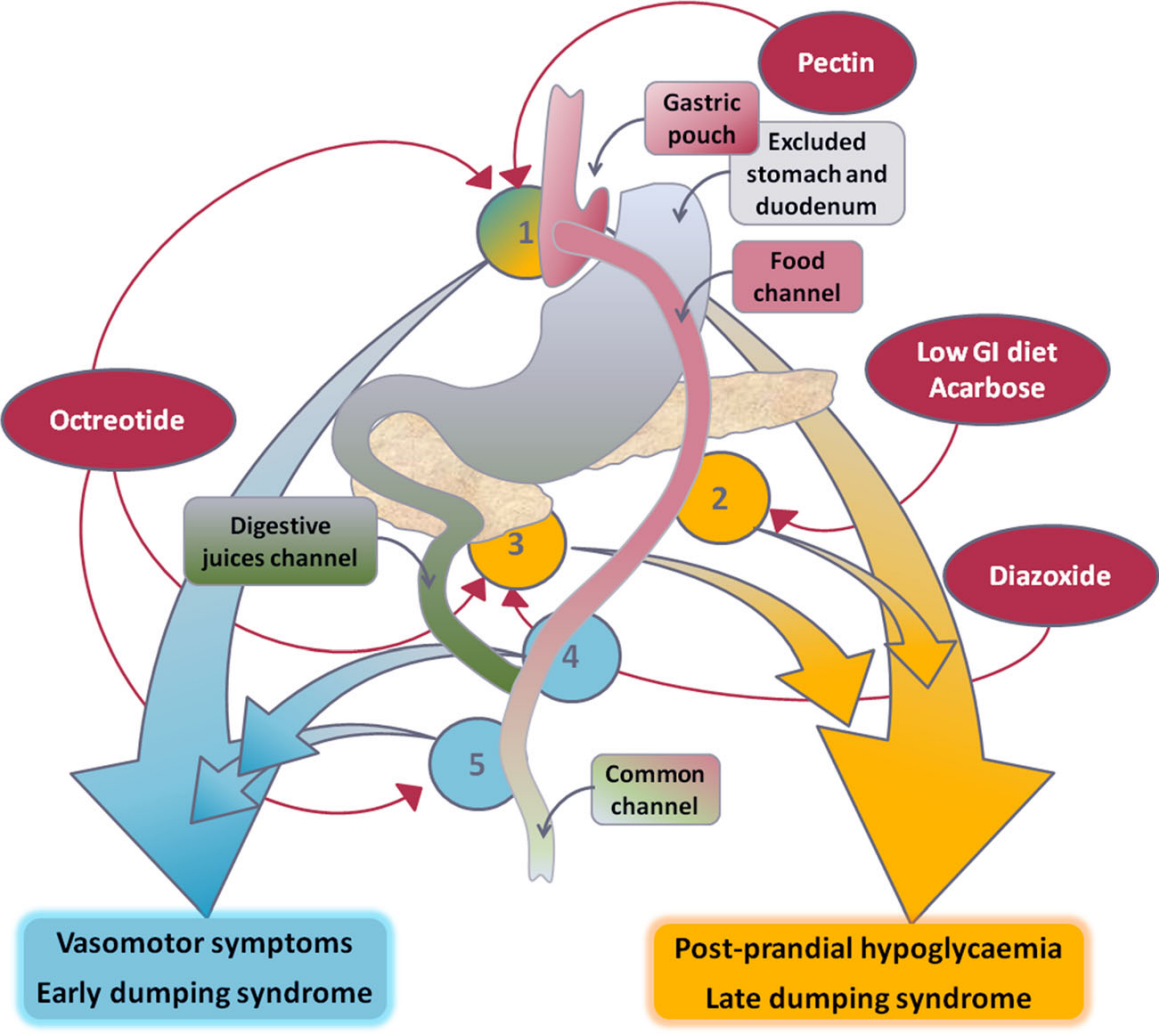
Pathophysiology of dumping syndromes following gastric bypass surgery and mode of action of common therapeutic agents. Reduced gastric volume results in rapid gastric emptying (*1*) and rapid glucose absorption (*2*), which induces a hyperinsulinemic response (*3*), leading to reactive hypoglycemia 1–3 h after food (late dumping syndrome). Rapid delivery of hyperosmolar chyme to the upper small bowel (*4*) induces release of several vasoactive gut hormones (*5*), such as vasoactive intestinal peptide, neurotensin, glucagon-like peptide-1 (GLP-1) and glucagon-dependent insulinotropic peptide (GIP), which results in vasomotor symptoms 10–60 min after food (early dumping syndrome). Agents that increase meal viscosity, such as pectin or guar gum, may help to slow down gastric emptying. The α-glucosidase inhibitor, acarbose, can slow down the breakdown and absorption of food sugars. Diazoxide inhibits insulin release from β cells in the pancreatic islets and can attenuate the hyperinsulinemic response. The somatostatin analogue, octreotide, works at multiple levels in the upper gastrointestinal tract, including slowing of gastric emptying and inhibition of secretion and release of insulin and vasoactive gut hormones, and can be useful in the treatment of intractable symptoms of both types of dumping syndrome

Early dumping occurs within an hour after food and is attributed to accelerated transit of ingested meals causing osmotic shifts in the small bowel and release of vasoactive intestinal hormones, leading to vasomotor symptoms such as flushing, palpitation, perspiration, tachycardia, hypotension and syncope (Fig. 1). It is frequently accompanied by gastrointestinal symptoms such as nausea, abdominal pain, borborygmi, bloating and diarrhoea.

##### Management of Early Dumping

Dietary measures include advising patients to consume smaller amounts in one sitting by dividing the recommended daily energy intake between six meals [33]. Patients are also advised to delay any liquid intake until at least 30 min after a meal. Lying down for 30 min after meals can prolong gastric emptying and help to reduce vasomotor symptoms.

#### Late Dumping Syndrome

Late dumping or postprandial hypoglycemia occurs 1 to 3 h after meals. It is partly incretin-mediated and is thought to occur in response to hyperinsulinemia following rapid glucose transit into the jejunum (Fig. 1). This results in a reactive hypoglycemia manifesting as diaphoresis, tremulousness, poor concentration, altered consciousness, palpitation and syncope.

The physiological increase in insulin secretion and insulin sensitivity that occurs in early gestation increases the risk of hypoglycemia in women who conceive following bariatric surgery. It is worth noting in this context that the standard oral glucose tolerance test used to identify gestational diabetes is frequently poorly tolerated in women who have had gastric bypass, sleeve gastrectomy or biliopancreatic diversion procedures as the rapid osmotic load of the glucose drink in the small intestine can cause significantly distressing symptoms from early dumping as well as profound reactive hypoglycemia from late dumping. Assessment of home capillary blood glucose (cBG) profiles and/or continuous glucose monitoring (CGM) over 1–2 weeks around 24–28 weeks of gestation may offer an alternative means of diagnosing gestational diabetes that is better tolerated in this context.

##### Management of Late Dumping

Dietary modifications are the mainstay of management of postprandial hypoglycemia and principally include the avoidance of refined carbohydrates in favour of low glycemic index foods. All rapidly absorbable carbohydrates (for example, all sweet or sweetened foods) should be eliminated from the diet to prevent late dumping symptoms [33]. A limited number of pharmacological agents have been used in the treatment of postprandial hypoglycemia in non-pregnant individuals [33]. Adding pectin or guar gum to increase the viscosity of food, which slows down gastric emptying, can ameliorate symptoms in the short term [33]; however, they are poorly tolerated due to unpalatability.

##### Diazoxide

Diazoxide decreases insulin release by inhibiting voltage-sensitive calcium channels [33]. It has been used clinically in the medical treatment of insulinoma, and administration of 100 to 150 mg three times daily for late dumping symptoms has been reported anecdotally. However, reproduction studies have revealed increased foetal resorptions, foetal skeletal anomalies and delayed parturition in rats, and skeletal and cardiac teratogenic effects in rabbits. It also crosses the placental barrier in animals and causes degeneration of foetal pancreatic β cells. Safety in pregnancy has not been established and the United States Federal Drug Agency (FDA) has classed diazoxide in *Pregnancy Category C* (animal reproduction studies have shown an adverse effect on the foetus, and there are no adequate and well-controlled studies in humans, but potential benefits may warrant use of the drug in pregnant women despite potential risks) [34]. Diazoxide appears in cord blood when given to pregnant women and may produce various foetal or neonatal adverse effects including hyperbilirubinemia, thrombocytopenia, altered carbohydrate metabolism, alopecia and hypertrichosis lanuginosa.

##### Octreotide

Somatostatin analogues, of which octreotide is the most commonly studied, act by decreasing gastric emptying rate, retarding transit through the small bowel, inhibiting release of gastrointestinal hormones, inhibiting insulin secretion and inhibiting postprandial vasodilation [33]. The major disadvantages of somatostatin analogues include the need for subcutaneous injections (which can be particularly problematic with short-acting preparations), adverse effects such as pain at injection sites, steatorrhea and gallstone formation and substantial cost. Whilst successful long-term treatment with somatostatin analogues in intractable postprandial hypoglycemia following gastric bypass surgery has been described [35], there are no adequate studies in pregnant women. Reproduction studies in rats and rabbits at doses up to 16 times the highest recommended human dose based on body surface area have shown no evidence of foetal harm due to octreotide. However, octreotide should be used during pregnancy only if clearly needed (FDA *Pregnancy Category B:* animal reproduction studies have failed to demonstrate a risk to the foetus, but there are no adequate and well-controlled studies in pregnant women) [36]. No congenital malformations have been reported in postmarketing data from a limited number of exposed pregnancies in women with acromegaly.

##### Acarbose

Acarbose, an intestinal α-glycosidase inhibitor, delays digestion and absorption of carbohydrates in the small intestine, and results in improved glucose tolerance, decreased release of gastrointestinal hormones and reduced incidence of hypoglycemia. Because of its mechanism of action, acarbose when administered alone does not cause hypoglycemia in the fasted state. Acarbose treatment often results in bloating, flatulence or diarrhoea, as the unabsorbed carbohydrates undergo bacterial fermentation in the small intestine [33]. Rodent studies of acarbose have shown no evidence of impaired fertility or harm to the foetus using the equivalent of nine times the exposure in humans based on blood drug levels. Studies in rabbits have shown no evidence of embryotoxicity at 10 times or teratogenicity at 32 times the dose in man based on body surface area. However, there are no adequate and well-controlled studies of acarbose in pregnant women (*FDA Pregnancy Category B*) [37]. Acarbose is a recognized treatment for postprandial hypoglycemia in non-pregnant individuals [33] and has separately been explored in gestational diabetes. Our *Case Study* (see box) is the first report in the published literature of the successful use of acarbose in postprandial hypoglycemia in pregnancy following bariatric surgery.

##### Continuous Enteral Feeding

Continuous enteral feeding via a nasogastric tube or a feeding jejunostomy to avoid dumping symptoms that are triggered by meal ingestion has been described in the management of refractory dumping syndrome [33]. However, this is a rather invasive intervention, with a major effect on daily life, and to our knowledge has not been reported in the management of dumping syndromes in pregnancy.

**Table:** 

Case study A 25-year-old woman with morbid obesity, acanthosis nigricans, polycystic ovarian syndrome, oligomenorrhoea and primary infertility and childhood-onset absence seizures underwent Roux-en-Y gastric bypass (RYGB) surgery. A year later, her weight had reduced from 125 to 78 kg and body mass index (BMI) from 47∙6 to 29∙7 kg/m2. This was accompanied by a remarkable resolution of acanthosis nigricans and restoration of normal menstruation, and she soon became pregnant spontaneously. She started experiencing symptomatic hypoglycemic episodes 1 to 3 h after food early in pregnancy. Fingerprick capillary blood glucose (cBG) monitoring and continuous glucose monitoring (CGM; MiniMed Paradigm 522, Medtronic®) confirmed non-fasting, postprandial hypoglycemia, with glucose levels of less than 2.2 mmol/L. Fasting plasma glucose was 4∙2 mmol/L, serum insulin 51 IU/L and C-peptide 418 pmol/L. Urine tested negative for sulphonylureas. Serum cortisol, thyroid hormones and vitamin and micronutrient levels were satisfactory. Despite dietary management with a low glycemic index (GI) diet postprandial hypoglycemia increased in severity and frequency, raising concerns of the risk of maternal neuroglycopenia and foetal hypoglycemia. After careful consideration of further options of management, she was commenced on acarbose in incremental doses up to 100 mg three times daily in the second trimester. Acarbose was well tolerated with significant reduction in hypoglycemia frequency and severity on cBG and CGM. She proceeded to term and delivered a healthy girl who is developing normally. Magnetic resonance imaging of the pancreas postdelivery revealed no abnormalities, and there was no further significant hypoglycemia on cBG and CGM. She went on to have a second pregnancy 2 years later. She again experienced hypoglycemic episodes, typically associated with consumption of high GI foods but not with low GI foods. The hypoglycemia was not as severe or as frequent as in the first pregnancy and was managed by low GI diet without resorting to acarbose. She proceeded to term and delivered a healthy boy who is also developing normally.	

### Nutrient Deficiencies

It is important to optimize the nutritional status of women prior to and following bariatric surgery [2, 38]. Nutritional deficiencies may vary depending on the type of surgery. For example, nutrient deficiencies may be minimal after gastric banding, which is a primarily restrictive procedure. Gastric bypass, the most common type of mixed restrictive and malabsorptive bariatric surgery worldwide, may be associated with deficiencies of iron, vitamin B_12_, calcium, vitamin D and other fat soluble vitamins, and trace elements. Sleeve gastrectomy, a predominantly restrictive procedure, is associated with the above deficiencies to a lesser extent. The less common duodenal switch and biliopancreatic diversion procedures are associated with significant malabsorption of macronutrients including protein and fat, and micronutrients and vitamins such as vitamins A, D and B_12_, calcium, iron, selenium, zinc and copper. It is crucial that people who have undergone bariatric surgery, women of childbearing age in particular, have dietary supplementation of multivitamins and micronutrients and specialist review regularly for monitoring of deficiencies [38, 39]. Care must be taken to ensure that any prescribed supplements and medications for women planning a family are safe in pregnancy. Retinol-based vitamin A products are best avoided in the first 12 weeks of pregnancy due to their teratogenic potential. For women found to have vitamin A deficiency, the beta carotene form of vitamin A is preferred. Protein intake should be maintained above 60 g per day [38, 40], and caloric restriction and active weight loss during pregnancy are not advisable even if overweight or obese. Gastric bands may need to be partially or completely deflated to allow appropriate nutritional intake and ameliorate pregnancy-related gastrointestinal symptoms.

### Surgical Complications

During the antenatal period, care must be taken to distinguish complications related to bariatric surgery from physiological manifestations of pregnancy. A history of bariatric surgery should not influence the course of labour and delivery. However, whilst bariatric surgery is not in itself an indication for caesarean section, rates of caesarean section do appear to be higher than average in women who have had bariatric surgery. Whilst nausea, vomiting and abdominal pain are common in normal pregnancies, in the context of bariatric surgery they may also occasionally represent surgical complications of prior bariatric surgery.

Notable surgical complications during pregnancy of previous weight loss procedures could include small bowel obstruction, internal hernias, gastric band erosion or migration and cholelithiasis [29, 41]. Small bowel obstruction is a well-recognized life-threatening late, albeit rare, complication of bariatric surgery, principally Roux-en-Y gastric bypass and commonly results from internal hernias and sometimes from volvulus or intussusceptions [4, 26]. The clinical presentation of early small bowel obstruction without bowel necrosis can be subtle [26]. Patients typically complain of crampy pain in the left upper quadrant or epigastric region that may radiate to the back. Vital signs, physical examination (barring tenderness over the area of pain) and laboratory studies may be normal. The generally mild signs and symptoms may be mistaken for common and benign pregnancy-related complaints. Thus, the patient’s surgical history of bariatric surgery and high clinical index of suspicion are required for early detection of small bowel obstruction. Tachycardia, abdominal distension, and elevated white blood cell counts, liver enzymes, amylase or lipase levels should raise concerns of bowel necrosis or perforation. When a pregnant patient with a history of bariatric surgery and abdominal complaints fails to respond as expected to appropriate treatment of the presumptive cause, she should be expeditiously reassessed, preferably in a centre with specialist bariatric expertise.

## Conclusion

In summary, bariatric surgery in women of childbearing age can present clinical challenges in subsequent pregnancies. Although rates of adverse maternal and foetal outcomes in obese women are reduced after bariatric surgery, pregnancy is best avoided for 12–24 months to reduce the risk of intrauterine growth retardation. Dumping syndromes occur not infrequently and need to be distinguished from common symptoms of a normal pregnancy, and specialist bariatric expertise should be sought where required. Dietary measures are the mainstay of management of dumping syndromes but pharmacotherapy may sometimes become necessary. Acarbose is the least hazardous pharmacological option for the management of late dumping/postprandial hypoglycemia in pregnancy. Nutrient deficiencies may vary depending on the type of surgery; it is important to optimize the nutritional status of women prior to and during pregnancy, including adequate protein and calorie intake and supplementation of vitamins and micronutrients. Finally, early diagnosis of surgical complications of prior weight loss procedures during pregnancy requires a high clinical index of suspicion and prompt referral to a specialist bariatric centre.
